# Supplementary material for: Mutations in PCYT2 disrupt etherlipid biosynthesis and cause a complex hereditary spastic paraplegia
Source: Brain. 2019 Oct 22;142(11):3382–97. doi: 10.1093/brain/awz291 (PMC6821184; doi:10.1093/brain/awz291)
Supplement: awz291_Supplementary_Data [file awz291_supplementary_data.zip › awz291-Suppl_data/Supplementary_Data4.zip!/Supplementary information 4 - plasma lipidomics.zip!/Plasma lipidomics/Control vs Patient3a/Statistical plots and data/PC[O] species/Volcano plot_alternative.pdf]

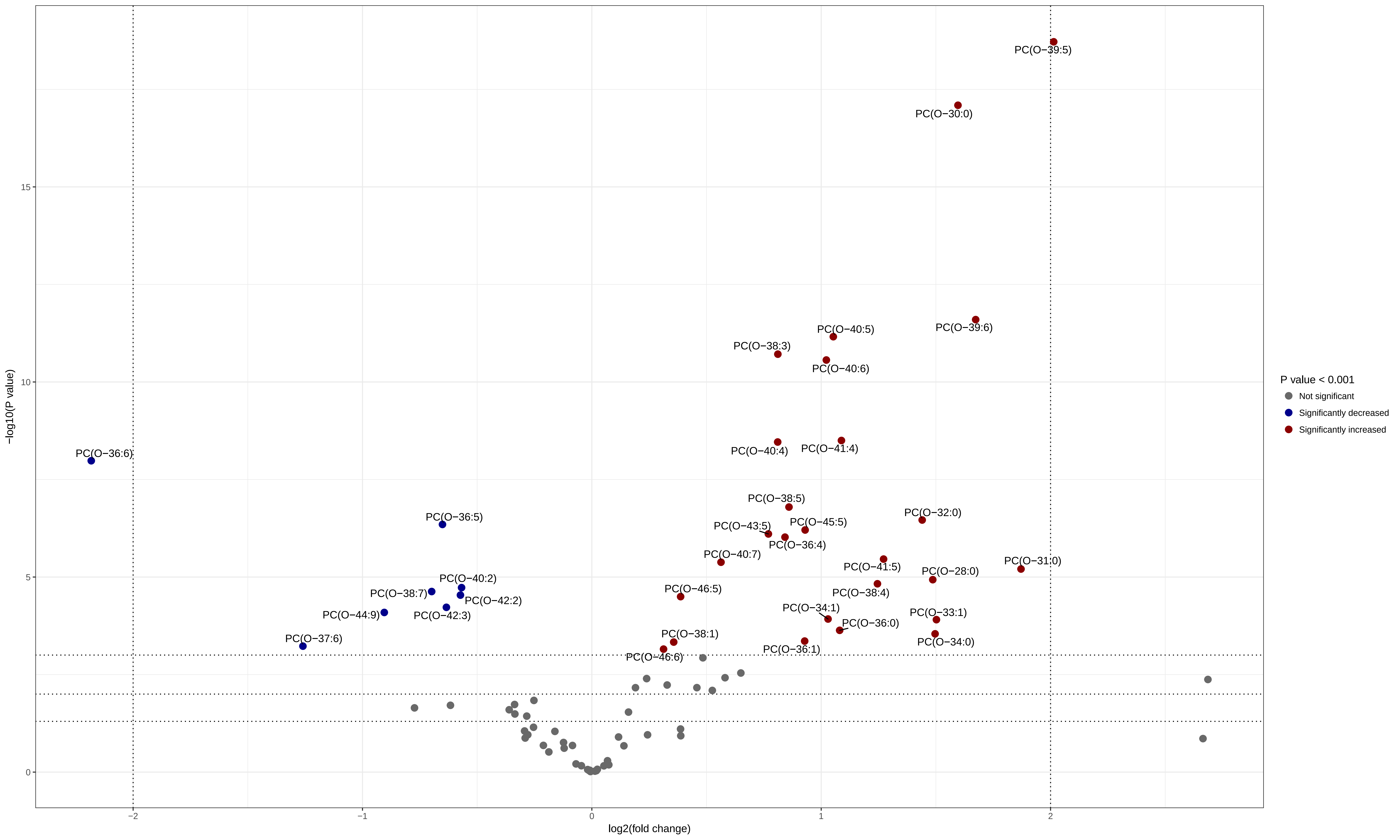

PC(O−39:5)
PC(O−30:0)
PC(O−39:6)
PC(O−40:5)
PC(O−38:3)
PC(O−40:6)
PC(O−40:4)
PC(O−41:4)
PC(O−36:6)
PC(O−38:5)
PC(O−32:0)
PC(O−45:5)
PC(O−43:5)
PC(O−36:4)
PC(O−36:5)
PC(O−40:7)
PC(O−31:0)
PC(O−41:5)
PC(O−28:0)
PC(O−40:2)
PC(O−46:5)
PC(O−38:7)
PC(O−42:2)
PC(O−38:4)
PC(O−34:1)
PC(O−33:1)
PC(O−44:9)
PC(O−42:3)
PC(O−36:0)
PC(O−38:1)
PC(O−37:6)
PC(O−34:0)
PC(O−36:1)
PC(O−46:6)
−log10(P value)
log2(fold change)
15
10
5
0
−2
−1
0
1
2
P value < 0.001
Not significant
Significantly decreased
Significantly increased
